# Insecticide‐contaminated honeydew: risks for beneficial insects

**DOI:** 10.1111/brv.12817

**Published:** 2021-11-21

**Authors:** Miguel Calvo‐Agudo, John F. Tooker, Marcel Dicke, Alejandro Tena

**Affiliations:** ^1^ Centro de Protección Vegetal y Biotecnología Instituto Valenciano de Investigaciones Agrarias (IVIA) Carretera de Moncada‐Náquera Km. 4,5 46113 Moncada Valencia Spain; ^2^ Laboratory of Entomology Wageningen University PO Box 16 6700AA Wageningen The Netherlands; ^3^ Department of Entomology The Pennsylvania State University University Park PA 16802 U.S.A.

**Keywords:** systemic insecticides, neonicotinoid, hemipteran, environmental risk assessment, pollinators, parasitic wasps, predators, invasive species

## Abstract

Honeydew is the sugar‐rich excretion of phloem‐feeding hemipteran insects such as aphids, mealybugs, whiteflies, and psyllids, and can be a main carbohydrate source for beneficial insects in some ecosystems. Recent research has revealed that water‐soluble, systemic insecticides contaminate honeydew excreted by hemipterans that feed on plants treated with these insecticides. This contaminated honeydew can be toxic to beneficial insects, such as pollinators, parasitic wasps and generalist predators that feed on it. This route of exposure has now been demonstrated in three plant species, for five systemic insecticides and four hemipteran species; therefore, we expect this route to be widely available in some ecosystems. In this perspective paper, we highlight the importance of this route of exposure by exploring: (*i*) potential pathways through which honeydew might be contaminated with insecticides; (*ii*) hemipteran families that are more likely to excrete contaminated honeydew; and (*iii*) systemic insecticides with different modes of action that might contaminate honeydew through the plant. Furthermore, we analyse several model scenarios in Europe and/or the USA where contaminated honeydew could be problematic for beneficial organisms that feed on this ubiquitous carbohydrate source. Finally, we explain why this route of exposure might be important when exotic, invasive, honeydew‐producing species are treated with systemic insecticides. Overall, this review opens a new area of research in the field of ecotoxicology to understand how insecticides can reach non‐target beneficial insects. In addition, we aim to shed light on potential undescribed causes of insect declines in ecosystems where honeydew is an important carbohydrate source for insects, and advocate for this route of exposure to be included in future environmental risk assessments.

## I INTRODUCTION

**Fig 1:**
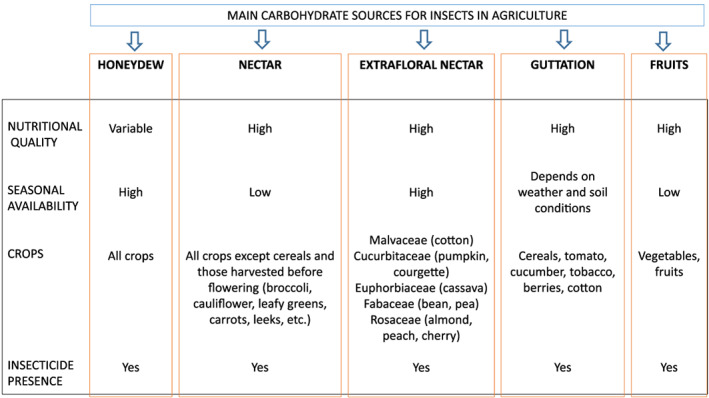
Main carbohydrate sources for beneficial insects in agriculture, their nutritional quality for insects and seasonal availability, the main crop groups in which they are present, and the presence of systemic insecticides or their residues in the carbohydrate source. Based on data from Wäckers, van Rijn & Heimpel (2008), Lundgren (2009), Tena *et al*. (2016), and Girolami *et al*. (2009).

Honeydew is the sugar‐excretion product of hemipterans, such as aphids, coccids, whiteflies, and psyllids, that feed on plants. This sugar source is exploited by many beneficial insects including bees, hoverflies, ants, parasitic wasps and predators (Hölldobler & Wilson, 1990; Lee, Andow & Heimpel, 2006; Hogervorst, Wäckers & Romeis, 2007; Konrad *et al*., 2009; Tena *et al*., 2013*b*; Calabuig *et al*., 2015; Meiners *et al*., 2017; Cameron, Corbet & Whitfield, 2019). Compared to other carbohydrate sources present in agricultural lands and some forests (Fig. 1), honeydew is highly accessible and can be abundant in nearly all crops and seasons (Lundgren, 2009). Notably, it was recently demonstrated that honeydew can contain insecticides that can negatively influence beneficial insect species (Calvo‐Agudo *et al*., 2019, 2020, 2021). More specifically, it was shown that hemipterans feeding on plants treated with systemic insecticides (i.e. water‐soluble insecticides that can move within plant vascular tissue) excreted honeydew laden with the active ingredient of the insecticides or its metabolites, and the honeydew was toxic to insects that consumed it (Calvo‐Agudo *et al*., 2019, 2020, 2021; Quesada, Scharf & Sadof, 2020).

Honeydew as route of exposure to water‐soluble insecticides has now been demonstrated for four species of honeydew producers belonging to four different families of hemipterans, five systemic insecticides with four different modes of action and translocation routes, and three plant species (Calvo‐Agudo *et al*., 2019, 2020, 2021; Quesada *et al*., 2020). This route of exposure, therefore, is likely to be common in agroecosystems where water‐soluble and systemic insecticides are used. The aim of this perspective paper is to discuss the relative importance of this pathway. First, we identify the potential routes through which honeydew can be contaminated with insecticides. Second, we discuss which hemipteran families are more likely to excrete contaminated honeydew. Third, we provide a list of systemic insecticides with different modes of action that might contaminate honeydew. Finally, we select several scenarios (model crop species and hemipterans) for which contaminated honeydew could be problematic for beneficial organisms. The crop species were selected because they have high economic importance in the EU and/or the USA, are commonly treated with systemic insecticides, and honeydew can be the main carbohydrate source for beneficial insects in fields of these crops.

## II HOW CAN SYSTEMIC INSECTICIDES REACH HONEYDEW?

**Fig 2:**
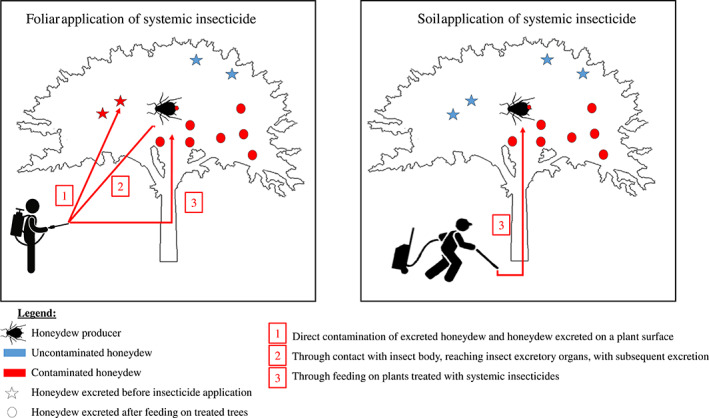
Three pathways by which honeydew can be contaminated with insecticides.

**Fig 3:**
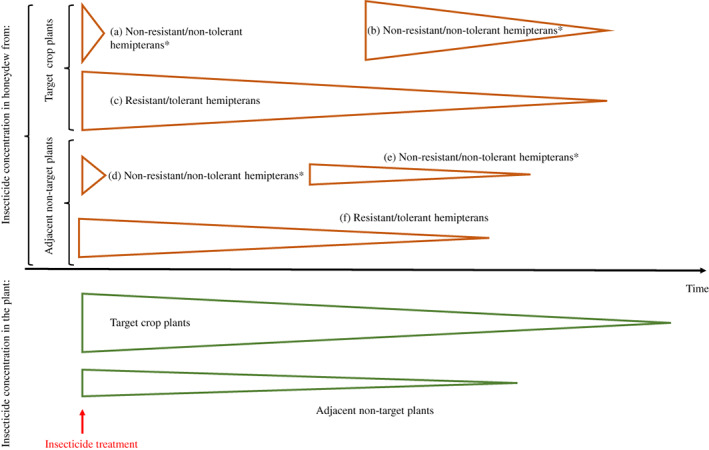
Proposed scenarios through which plant‐incorporated insecticides can reach honeydew excreted by hemipterans. The width of triangles represents insecticide concentration in the plant (green) and in honeydew (orange) after an insecticide treatment (red arrow). *Resistant/tolerant hemipterans can occur here too.

Broadly, water‐soluble systemic insecticides might reach honeydew through three different pathways (Fig. 2). (*i*) *Direct contamination of honeydew*: honeydew already present on a plant can be contaminated by the direct spraying of insecticides (Fig. 2). (*ii*) *Through insects that excrete honeydew*: insecticides can be directly absorbed into the body of honeydew producers when they are sprayed, and honeydew producers could then excrete the insecticide *via* their honeydew (Fig. 2). (*iii*) *Through plants and honeydew producers*: systemic insecticides are translocated to all parts of the plant, and honeydew producers that feed on treated plants can excrete the insecticide *via* their honeydew (Calvo‐Agudo *et al*., 2019) (Fig. 2). Systemic insecticides are applied using at least six techniques: spraying, soil drenching, injection into the plant (mostly for tree crops), as granules, drip irrigation (chemigation) or as seed coatings. When systemic insecticides are applied by spraying (Calvo‐Agudo *et al*., 2019, 2020), all three exposure pathways are likely to take place. By contrast, if systemic insecticides are applied to the soil, in the irrigation system, injected into the trunk, as granules or used as seed coatings, only the third pathway can occur (Calvo‐Agudo *et al*., 2019, 2020, 2021). Within this third pathway, systemic insecticides might reach honeydew under several possible scenarios (Fig. 3), which are considered in turn below.

### (1) Through hemipterans that feed on treated crop plants

#### (a) Non‐tolerant/non‐resistant hemipterans excrete contaminated honeydew before they die

Non‐tolerant/non‐resistant honeydew producers are able to excrete contaminated honeydew during short periods of time, before they die as a consequence of ingesting insecticide (route a in Fig. 3). This scenario might occur from a few hours after insecticide application until hemipterans die due to the treatment. For instance, the mealybug *Planococcus citri* (Risso) (Hemiptera: Pseudococcidae) and the soft scale *Toumeyella pini* (King) (Hemiptera: Coccidae) excrete honeydew contaminated with systemic insecticides for up to 5–8 days following treatment (see supporting information in Calvo‐Agudo *et al*., 2019; Quesada *et al*., 2020). The period over which such non‐tolerant/non‐resistant honeydew producers can excrete honeydew is likely to depend on the mode of action of the insecticide and its physicochemical profile, mode of application, plant species, and honeydew‐producer species.

#### (b) Non‐tolerant/non‐resistant hemipterans excrete contaminated honeydew once insecticide concentration decreases in the plant

Non‐tolerant/non‐resistant hemipterans can also recolonize insecticide‐treated plants after the insecticide concentration has diminished to levels allowing their survival on the host plant (b in Fig. 3). We expect this scenario to be common because current agriculture is dominated by extensive monoculture crops whose seeds are commonly coated with systemic insecticides. The seeds of cereals, soybean (*Glycine max* L.), cotton (*Gossypium* spp.), sunflower (*Helianthus annuus* L.) or rapeseed (*Brassica napus napus* L.) are coated with neonicotinoids everywhere except in the EU, which represents less than 4% of the world agricultural landscape (Worldbank, 2020). The protection period for systemic insecticides applied to seeds is often limited to just a few weeks (Alford & Krupke, 2017); plants can become reinfested with honeydew producers when insecticide concentrations decrease and these colonizers could then excrete contaminated honeydew (Calvo‐Agudo *et al*., 2021). The same scenario might occur when insecticides are sprayed or applied to the soil or injected into the trunk and honeydew producers are able to tolerate variable concentrations. In addition to a decrease in insecticide concentration in the plant over time, this second scenario could arise when systemic insecticides are sprayed, but the target plant receives lower insecticide volumes due to incorrect application or unfavourable weather conditions.

#### (c) Tolerant/resistant hemipterans excrete contaminated honeydew while feeding on treated plants until the insecticide is completely degraded

Another common scenario is when honeydew producers are resistant or tolerant to the active ingredient (c in Fig. 3). This scenario is likely to occur when the tolerant/resistant honeydew producer is not the target species of the insecticide application or when the target species has developed resistance. As one example of a tolerant species, mealybugs are tolerant to the active ingredients flonicamid and pymetrozine (El‐Zahi, El‐Salam Aref & Mohammad Korish, 2016; Nagrare *et al*., 2016; Barbosa *et al*., 2018; Rezk *et al*., 2019) that are selectively used to protect numerous crops against aphids (Belchim, 2020; Syngenta, 2020). Aphids coexist with mealybugs in citrus plants, where these active ingredients are applied (Pekas *et al*., 2011; Tena, Llácer & Urbaneja, 2013*a*; Tena *et al*., 2013*b*). The mealybug *P. citri* excretes contaminated honeydew when citrus trees are sprayed with either flonicamid or pymetrozine (Calvo‐Agudo *et al*., 2020). Honeydew contaminated with these insecticides harmed the hoverfly *Sphaerophoria rueppellii* (Wiedemann) (Diptera: Syrphidae). Excretion of contaminated honeydew by resistant hemipteran species has yet to be demonstrated; nevertheless, one study showed that individuals of *Nilaparvata lugens* Stal (Homoptera: Delphacidae) that were resistant to fipronil excreted honeydew during the 30 days that the experiment lasted (Ling *et al*., 2009). Even though this study did not measure the concentrations of fipronil in honeydew, it seems likely that honeydew would be contaminated with fipronil. Other key pest species such as, silverfleaf whitefly *Bemisia tabaci* (Gennadius) (Hemiptera: Aleyrodidae) and green peach aphid *Myzus persicae* (Sulzer) (Hemiptera: Aphididae), have developed resistance to more than 40 and 70 active ingredients, respectively, some of which are used systemically in plants (van Leeuwen *et al*., 2010; Insecticide Resistance Action Committee, 2020). To our knowledge, at least 24 species of hemipterans that excrete honeydew are tolerant or have potential to develop resistance to different systemic insecticides (see online Supporting Information, Table S1). It is important to highlight that tolerant/resistant hemipterans can excrete contaminated honeydew from a few hours after the treatment until these insecticides or their metabolites are completely degraded in the plant. Therefore, we expect that tolerant/resistant hemipterans excrete contaminated honeydew for a longer period of time than non‐tolerant/non‐resistant hemipteran species (Fig. 3).

### (2) Through hemipterans that feed on non‐target plants

Before systemic insecticides degrade, they can be transported to adjacent crops, co‐occurring weeds, field‐side vegetation, or adjacent habitats or ecosystems by movement in water or insecticide drift (Greatti *et al*., 2006; Krupke *et al*., 2012; Goulson, 2013; Hladik, Kolpin & Kuivila, 2014; Pearsons *et al*., 2021) (d–f in Fig. 3). During these movements, systemic insecticides can reach non‐target plants, even at concentrations exceeding those of the treated crop (Botías *et al*., 2015). Once insecticides have been absorbed by non‐target plants, they can be ingested and excreted by hemipterans *via* the pathways described above for target plants (a–c in Fig. 3).

## III HEMIPTERAN SPECIES LIKELY TO EXCRETE CONTAMINATED HONEYDEW

**Fig 4:**
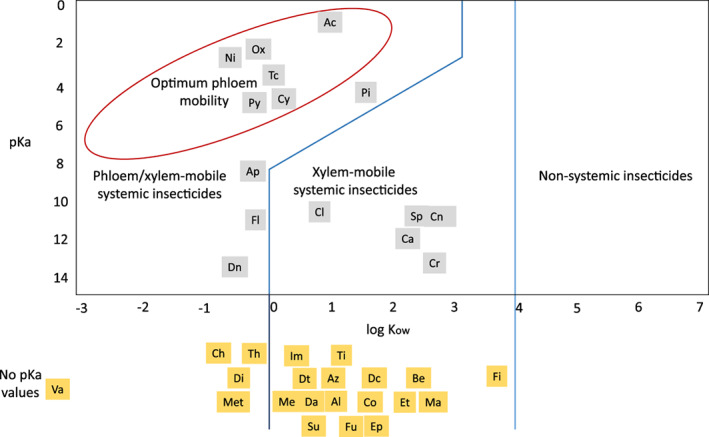
Behaviour of systemic insecticides according to their mobility in plants. pKa, dissociation constant; log K_ow_, octanol/water‐partition coefficient Adapted from Bromilow *et al*. (1990) and University of Hertfordshire (2021). Yellow boxes represent insecticides that have missing pKa values or that the insecticide cannot be dissociated. Ac, Acetamiprid; Al, Aldicarb; Ap, Acephate; Az, Azametiphos; Be, Benzoximate; Ca, Carbaryl; Ch, Cartap hydrochloride; Cl, Clothianidin; Cn, Chlorantraniliprole; Co, Carbofuran; Cr, Chromafenozide; Cy, Cyromazine; Da, Dazomet; Dc, Dichlorvos; Di, Dicrotophos; Dn, Dinotefuran; Dt, Dimethoate; Ep, Ethiprole; Et, ethiofencarb; Fi, Fipronil; Fl, Flonicamid; Fu, Flupyradifuron; Im, Imidacloprid; Ma, Malathion; Me, Methomyl; Mt, Methamidophos; Ni, Nitenpyran; Ox, Oxamyl; Pi, Pirimicarb; Py, Pymetrozine; Sp, Spirotetramat; Su, Sulfoxaflor; Th, Thiamethoxam; Ti, Thiacloprid; Tc, Thiocyclam; Va, Vamidothion.

The feeding behaviour of hemipterans might also affect excretion of contaminated honeydew. For instance, whiteflies feed mostly on plant phloem (Lei, Tjallingii & Lenteren, 1997); therefore, they will rarely excrete honeydew contaminated with insecticides that move through the xylem (Bromilow, Chamberlain & Evans, 1990). On the other hand, mealybugs, aphids and psyllids feed frequently on both phloem and xylem and thus might excrete insecticides that move through either vessel (Spiller, Koenders & Tjallingii, 1990; Cen *et al*., 2011; Obok, Wetten & Allainguillaume, 2018). Under field conditions, mealybugs and whiteflies excrete the systemic insecticide pymetrozine, which moves through both xylem and phloem, but only mealybugs excrete flonicamid, an insecticide that moves through the xylem (Fig. 4; Calvo‐Agudo *et al*., 2020).

## IV SYSTEMIC INSECTICIDES LIKELY TO CONTAMINATE HONEYDEW

Translocation properties of systemic insecticides might also affect honeydew contamination. These properties include the water solubility, the capacity of insecticides to dissolve in lipophilic (non‐aqueous) solutions, measured as the octanol/water‐partition coefficient (log K_ow_), and the charge of their molecules at different pHs, measured as the dissociation constant (pKa) (Bromilow *et al*., 1990; Sánchez‐Bayo, Tennekes & Goka, 2013). These properties are used to classify insecticides according to their mobility in plants (Fig. 4). Here, we summarize some of the main groups of systemic insecticides that are more likely to contaminate honeydew according to: the different contamination pathways (Fig. 3), their mobility in phloem and xylem (Fig. 4), and their persistence in the environment. These insecticides comprise the following groups: (*i*) neonicotinoids and sulfoximines; (*ii*) flonicamid pyridine azomethine derivatives, (*iii*) tetramic and tetronic acid derivatives; (*iv*) diamides; (*v*) phenylpyrazoles; and (*vi*) carbamates and organophosphates. Other groups such as cyromazine, diacylhydrazines (chromafenozide) and methyl isothiocyanate generators (dazomet) are also likely to contaminate honeydew, but have been excluded herein because we found little published information concerning them.

In addition to insecticides, some fungicides and herbicides are highly mobile in water and persistent in the environment (Gavrilescu, 2005; University of Hertfordshire, 2021). These active ingredients might also reach honeydew excreted by hemipterans and could be toxic to beneficial insects or synergize the toxicity of insecticides if both reach the honeydew. For instance, mesotrione and atrazine are two herbicides that might be toxic through this route. Both herbicides are highly water‐soluble with an optimum phloem mobility (University of Hertfordshire, 2021), and in combination they cause sublethal effects on workers of the pollinator *Partamona helleri* Friese (Hymenoptera: Apidae) that feed on it (dos Santos Araujo, Bernandes & Martins, 2021). Similarly, the oral toxicity of some systemic insecticides is synergized by propiconazole, a water‐soluble fungicide with some degree of mobility through the plant phloem (Sgolastra *et al*., 2017; Tosi & Nieh, 2019; University of Hertfordshire, 2021). Therefore, this fungicide, and others with similar modes of action and physiochemical properties, may reach the honeydew excreted by some hemipteran species. This review focuses on how insecticides might reach honeydew and affect beneficial insects; however, we note these examples to highlight that other pesticides might reach hemipteran honeydew and should be studied in greater detail.

### (1) Neonicotinoids and sulfoximines

Neonicotinoids and sulfoximines are systemic insecticides that bind to the acetylcholine site on nicotinic acetylcholine receptors (nAChRs), causing a range of symptoms from hyper‐excitation to lethargy and paralysis (Insecticide Resistance Action Committee, 2020). Neonicotinoid insecticides were used extensively over recent decades because they were considered economic, highly effective against a broad spectrum of insect pests, and could be applied in different modes: foliar spray, soil drench, soil granules, injected into irrigation systems, injected directly into trees, or as a seed coating (Jeschke *et al*., 2011). However, neonicotinoids can be highly persistent in water, plants and soils, where they can remain for years (Table S2) (Byrne *et al*., 2014; Humann‐Guilleminot *et al*., 2019), and can be highly toxic to beneficial insects, especially pollinators (Pisa *et al*., 2015) (see oral median lethal dose LD_50_ values for honeybees in Table S2; LD_50_ is the dose required to cause death of 50% of a tested population after a specified test duration). Due to their high persistence and toxicity to beneficial insects, in 2018 the EU banned use of the neonicotinoids thiamethoxam, imidacloprid and clothianidin on outdoor crops (European Commission, 2018a, 2018b, 2018c). However, these three insecticides remain in use in most countries outside the EU. We expect neonicotinoids to reach non‐target insects when they feed on contaminated honeydew (Fig. 3).

Other neonicotinoids such as dinotefuran, thiacloprid, acetamiprid and nitenpyram are also expected to reach hemipterans due to their physicochemical properties and water solubility. They have already been found in plant reproductive tissues such as nectar and pollen (Mullin *et al*., 2010; Stoner & Eitzer, 2013), and may be toxic *via* oral exposure to non‐target beneficial insects (Claus *et al*., 2021).

The sulfoximine sulfoxaflor is a systemic insecticide used against hemipterans in a wide variety of crop species (Abdourahime *et al*., 2019). Sulfoxaflor is highly soluble in water and can be transported around plant tissues following foliar or seed application (Siviter, Brown & Leadbeater, 2018). Compared to neonicotinoids, however, it appears to have a relatively short half‐life in soil (~2.2 days) and plant tissues (~9 days) (EPA, 2016), reducing the period in which honeydew can become contaminated (Table S2) (European Food Safety Authority, 2014). Nevertheless, a risk assessment by the European Food Safety Authority (EFSA) indicated high acute oral risks to pollinators (European Food Safety Authority, 2014; Abdourahime *et al*., 2019; Siviter *et al*., 2020) (see oral LD_50_ values for honeybees in Table S2); therefore, we expect scenarios of honeydew contamination and toxicity similar to neonicotinoids but during shorter periods of time.

### (2) Flonicamid and pyridine azomethine derivatives

Flonicamid and pyridine azomethine derivatives such as pymetrozine are systemic insecticides with different modes of action, but both disrupt feeding and other behaviours in target insects (Belchim, 2020; Syngenta, 2020). Both insecticides can be soil or foliar applied against numerous pests such as whiteflies, aphids, planthoppers or leafhoppers (Belchim, 2020; Syngenta, 2020), but most mealybug and psyllid species survive exposure to these insecticides (Qureshi, Kostyk & Stansly, 2014; El‐Zahi *et al*., 2016; Rezk *et al*., 2019). Flonicamid and pymetrozine have high water solubility but their persistence in soil and plants is unclear (Table S2). For instance, under laboratory conditions flonicamid has a soil half‐life of 1.1 day (University of Hertfordshire, 2021), but 2.04–14.2 days in the field (Liu *et al*., 2014; Wang *et al*., 2018*a*). In plants, residues of flonicamid or its metabolites can be found in plants 6–21 days after application (Liu *et al*., 2014; Wang *et al*., 2018*a*). Tolerant mealybugs and psyllids might, therefore, excrete contaminated honeydew at least 21 days post‐application (b, d in Fig. 3). Compared to neonicotinoids, flonicamid and pymetrozine are less toxic to beneficial insects (see LD_50_ values in Table S2) (Calvo‐Agudo *et al*., 2019, 2020).

### (3) Tetramic and tetronic acids

The tetramic‐acid derivative spirotetramat inhibits lipid biosynthesis, leading to insect death (Insecticide Resistance Action Committee, 2020). Spirotetramat can be soil or foliar applied against scale insects, mealybugs, aphids, whiteflies, mites or thrips (Bayer Crop Science, 2020), and has medium mobility in soil, and a very short soil half‐life (0.19 days) (Table S2). However, some of its metabolites such as spirotetramat‐enol or spirotetramat‐ketohydroxy, exhibit higher mobility and persistence in soil (European Food Safety Authority, 2013*a*). For instance, the metabolite spirotetramat‐ketohydroxy has a half‐life of 1.5–14.2 days in soil. When applied to crops, spirotetramat and its metabolites can remain in plants for nearly 30 days at low concentrations (Chen *et al*., 2016). When applied, spirotetramat can be excreted at high concentrations through non‐resistant/non‐tolerant hemipteran honeydew during short periods of time (a in Fig. 3). For example, *T. pini* excreted honeydew contaminated with spirotetramat for at least 8 days after treatment, before they died from ingesting the insecticide (a in Fig. 3; Quesada *et al*., 2020). In addition, tolerant/resistant hemipterans, such as *B. tabaci* (Bielza *et al*., 2019), might excrete spirotetramat or its metabolites in their honeydew for long periods of time until the insecticide is degraded (b, d in Fig. 3). Compared to neonicotinoids, spirotetramat is less toxic to parasitic wasps, predators and pollinators (see LD_50_ values in Table S2) (Planes *et al*., 2013; Vanaclocha *et al*., 2013; European Food Safety Authority, 2013*a*).

### (4) Diamides

Diamides activate muscle ryanodine receptors, leading to contraction and paralysis. Ryanodine receptors mediate calcium release from intracellular stores into the cytoplasm (Insecticide Resistance Action Committee, 2020). The diamide chlorantraniliprole is used against lepidopterans and is highly water soluble and persistent (European Food Safety Authority, 2013*b*; Table S2). Residues of this insecticide are present in pollen and nectar for at least 8 days after foliar application (Kyriakopoulou *et al*., 2017). We expect chlorantraniliprole to be excreted in hemipteran honeydew for long periods due to its high persistence and water solubility when tolerant hemipterans, such as some species of aphids or whiteflies, feed on contaminated phloem (Barrania & Abou‐Taleb, 2014; Nagrare *et al*., 2016) (Table S1). Compared to neonicotinoids, diamides are less toxic to parasitic wasps, predators and pollinators (see LD_50_ values in Table S2). However, chlorantraniliprole was toxic to the parasitic wasp *Lysiphlebus testaceipes* (Cresson) (Hymenoptera; Braconidae) and the lacewing *Chrysoperla carnea* (Stephens) (Neuroptera: Chrysopidae) by oral exposure (Gontijo *et al*., 2014; Moscardini *et al*., 2014).

### (5) Phenyl‐pyrazoles

Phenylpyrazoles block the γ‐amino butyric acid (GABA)‐activated chloride channel, causing hyperexcitation and convulsions. GABA is the major inhibitory neurotransmitter in insects (Insecticide Resistance Action Committee, 2020). Fipronil is a highly persistent phenylpyrazole with some degree of water mobility (European Food Safety Authority, 2013*c*; Table S2). However, it is important to highlight that mobility in plants of some insecticides such as fipronil can increase with certain copolymers (a detailed review of this active ingredient can be found in Bonmatin *et al*., 2015). Consequently, fipronil has been detected in plant vegetative tissues and reproductive organs including nectar, pollen and fruits (European Food Safety Authority, 2013*c*). Fipronil is highly toxic to many orders of insects, including hemipterans (Pisa *et al*., 2015) (see oral LD_50_ values for honeybees in Table S2). Therefore, we expect that hemipterans might excrete fipronil in their honeydew at least for short periods of time (a in Fig. 3), which might be prolonged if they are resistant to this phenylpyrazole (Table S1).

### (6) Carbamates and organophosphates

Carbamates (CMs) and organophosphates (OPs) contain insecticides that inhibit acetylcholinesterase (AChE), causing hyperexcitation in insects, and some active ingredients within these two groups are systemic (Insecticide Resistance Action Committee, 2020). CMs and OPs are toxic to a broad range of insects (see LD_50_ value in Table S2), and their use has decreased because of their negative effects on invertebrates, birds, fish and mammals (Sánchez‐Bayo, 2012). OPs and CMs were routinely applied between 1960 and 2000 and, as a consequence, many hemipterans have developed resistance/tolerance to several active ingredients (Table S1). Most systemic OPs and CMs are highly soluble in water and their persistence in soil and plants varies from low to medium (Table S2). For instance, the CM pirimicarb and the OP dimethoate can remain in plants for 31 and 38 days, respectively (Szeto, Vernon & Brown, 1985). In general, OPs and CMs are highly toxic to many beneficial insects (Mommaerts & Smagghe, 2011). Pirimicarb and dimethoate are particularly likely to contaminate honeydew. Pirimicarb was found in more than 50% of the samples of surface water in regions of Buenos Aires (Argentina) and Southern Ontario (Canada) (Struger *et al*., 2016; Natale *et al*., 2018), and many aphids have developed resistance to it (Table S1). Similarly, dimethoate is a common insecticide applied to fields in the USA (van Scoy, Pennell & Zhang, 2016). More than 816 tons of dimethoate are applied annually mostly to wheat (*Triticum aestivum* L.), cotton, maize (*Zea mays* L.) and alfalfa (*Medicago sativa* L.). A study conducted on surface water from California detected dimethoate in 9% of the samples analysed, with a highest concentration of 11.5 ppb (van Scoy *et al*., 2016). Furthermore, many hemipteran species have developed resistance to it (Table S1). We therefore expect ample opportunities for beneficial insects to be exposed to these active ingredients when feeding on honeydew from hemipterans on treated plants.

## V POTENTIAL CROPS IN WHICH HONEYDEW CAN BE CONTAMINATED WITH SYSTEMIC INSECTICIDES

**Fig 5:**
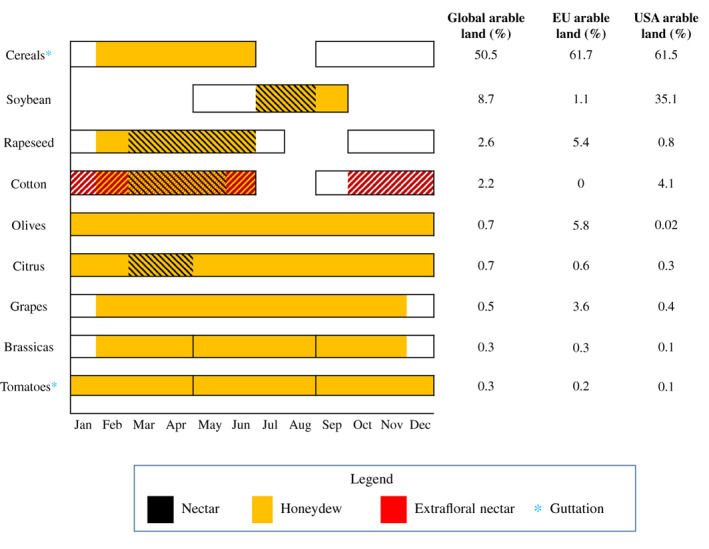
Assessment of risk of exposure of beneficial insects to honeydew contaminated with systemic insecticides for common crops in the USA and EU.

In 2018, the global total cropland area was more than 1431 Mha (FAOSTAT, 2018). Cropland area is about 87.3 Mha (6.1% of the total cropland surface) in the EU and 101 Mhas (7.1%) in the USA, and these areas contain several crop species in which honeydew is likely the main carbohydrate source for beneficial insects. We review these regions to emphasize the risk posed by insecticide‐contaminated honeydew (Fig. 5). The examples reviewed here can be extrapolated to other regions, crop species, hemipteran species, and insecticides.

### (1) Extensive crops

The cereals wheat, maize, rice (*Oryza sativa* L.), barley (*Hordeum vulgare* L.), sorghum (*Sorghum* spp.), rye (*Secale cereale* L.), oat (*Avena sativa* L.), millet [*Pennisetum glaucum* (L.) R. Br], and triticale (x *Triticosecale* Wittmack) occupy nearly 50.5% of the worldwide harvested area (723 Mha). In the EU and the USA, these crops represent 61.7% (53.9 Mha) and 61.5% (53.7 Mha) of the total agricultural land, respectively (Fig. 5). Cereals do not produce nectar, but guttation drops may appear on humid and windless days (Shawki *et al*., 2018; Urbaneja‐Bernat *et al*., 2020). These crops can be infested with many hemipterans that may provide honeydew during the growing season to beneficial insects; these hemipterans include aphid species [e.g. *Schizaphis graminum* (Rondani), *Diuraphis noxia* (Kurdjumov), *Sitobion avenae* (Fabr.), *Rophalosiphum maidis* (Fitch), *Ropalosiphum padi* (L.), *Aphis fabae* Scop, *M. persicae*, *Metopolophium dirhodum* (Wlk.), root‐aphids such as *Tetraneura nigriabdominalis* (Sasaki)] and mealybugs [*Brevannia rehi* (Lindinger)]. Therefore, depending on the surrounding landscape, honeydew might represent a main sugar source for beneficial insects in these agroecosystems. In fact, 59% of parasitic wasps and 44% of hoverflies collected in spring wheat fields had fed on honeydew, and 55% of parasitic wasps from winter wheat fields had fed on honeydew (Hogervorst *et al*., 2007). Furthermore, parasitic wasps captured in wheat fields throughout the year were found to have fed on honeydew (Luquet *et al*., 2021). In the EU, aphids can be treated with systemic insecticides, including acetamiprid, sulfoximines, spirotetramat, and flonicamid. In the USA, this list also includes several OPs, CMs, phenylpyrazoles, and neonicotinoids (Insecticide Resistance Action Committee, 2020). When systemic insecticides are sprayed and aphids are not resistant, they can excrete honeydew during short periods until they die from the insecticide (a in Fig. 3). However, when seeds are coated with neonicotinoids (b in Fig. 3), or aphids develop resistance to the insecticide (c in Fig. 3), the time frame in which they can excrete honeydew containing insecticides is likely to be longer.

Seeds of other herbaceous crop species, such as cotton, rapeseed or soybean, are commonly coated with neonicotinoids, such as imidacloprid, thiamethoxam or clothianidin. This is the leading delivery method of neonicotinoids throughout the world (Bonmatin *et al*., 2015). In the USA, more than 50% of soybeans and 52–77% of cotton, and 79–100% of maize hectares were sown with seeds coated with neonicotinoids in 2011 (Douglas & Tooker, 2015) and these amounts appear to continue to increase (Tooker, Douglas & Krupke, 2017; DiBartolomeis *et al*., 2019). For soybeans grown from seeds coated with thiamethoxam, the soybean aphid *Aphis glycines* Matsumura (Hemiptera: Aphididae) can colonize plants 25 days after sowing and excreted honeydew containing clothianidin, the derivate metabolite of thiamethoxam, for up to 42 days after sowing (Calvo‐Agudo *et al*., 2021). Honeydew is a common food source for parasitic wasps in soybean fields (Lee *et al*., 2006), so it seems likely that honeydew contaminated with neonicotinoids will be problematic for resident natural enemies, consistent with previous research that showed that natural enemies of the soybean aphid were susceptible to neonicotinoid‐contaminated honeydew (Calvo‐Agudo *et al*., 2021).

Cotton is an example in which three plant‐derived sugar sources for beneficial insects may be simultaneously contaminated with systemic insecticides (Fig. 5). Extrafloral nectar is the main food source because it has high nutritional quality and is available throughout the growing season (Limburg & Rosenheim, 2001), but it can also be contaminated with neonicotinoids (Jones *et al*., 2020). Floral nectar is available only during 4–6 weeks of the growing period, but can be contaminated by systemic insecticides (Jiang *et al*., 2018). In addition, honeydew excreted by the cotton aphid *Aphis gossypii* Glover (Hemiptera: Aphidae) can be present at variable quantities throughout the season (Gore *et al*., 2013; Zhou *et al*., 2014; University of California, 2020). Our research suggests that honeydew from *A. gossypii* is likely to be contaminated by neonicotinoids coated on seeds, or perhaps by other applications later in the season (Calvo‐Agudo *et al*., 2021). Despite honeydew representing a main food source for beneficial insects in cotton fields (Hagenbucher, Wäckers & Romeis, 2014), the ecological impacts of contamination of honeydew have been neglected.

### (2) Fruit crops

Citrus, grapes (*Vitis vinifera* L.) and olives (*Olea europaea* L.) are key crops of southern European and USA agriculture. For example, citrus crops occupy 17.45% of the global area used for fruit crop species of the worldwide harvested area (9.67 Mha). In the EU and the USA, citrus crops represent 510551 and 283591 ha, respectively (FAOSTAT, 2018). The flowering period lasts 2–3 months (Fig. 5), and permanent ground cover that provides additional nectar is scarce (Tena *et al*., 2013*b*; Gómez *et al*., 2018). However, a diverse and dynamic community of hemipterans feed on citrus and can excrete large quantities of honeydew throughout the year (Pekas *et al*., 2011; Tena *et al*., 2013*a*). In Mediterranean citriculture, there are numerous naturally controlled hemipterans that are often considered secondary pests and rarely need to be controlled chemically (Urbaneja *et al*., 2020). For instance, aphids (*Aphis spiraecola* Patch and *A. gossypii*) are highly abundant early in spring, coccids (*Coccus hesperidum* L. or *Saissetia oleae* Olivier) and mealybugs (*P. citri*) are dominant at the end of the spring and during summer, and whiteflies [*Aleurothrixus floccosus* (Maskell)] can be present on tender leaves in autumn (Pekas *et al*., 2011). Hence, honeydew is a commonly available food source for beneficial insects, including parasitic wasps of non‐honeydew‐producing herbivores in these agroecosystems (Tena *et al*., 2013*b*; Calabuig *et al*., 2015). Aphids or whiteflies can be treated with systemic insecticides such as sulfoxaflor, spirotetramat, acetamiprid or flonicamid when they exceed the economic injury level (Insecticide Resistance Action Committee, 2020; GIP Cítricos, 2021). While aphids or whiteflies can excrete these insecticides *via* honeydew for short periods of time (a in Fig. 3), or for longer if they develop resistance (c in Fig. 3), tolerant hemipterans such as *P. citri* can excrete contaminated honeydew for longer periods (c in Fig. 3) (Calvo‐Agudo *et al*., 2020).

In the citrus industry in Florida, USA, numerous broad‐spectrum systemic insecticides such as OPs, CMs, neonicotinoids, sulfoximines or spirotetramat are applied to control the Asian citrus psyllid, *Diaphorina citri* Kuwayama (Qureshi *et al*., 2014). This psyllid, which excretes honeydew (Ammar *et al*., 2013), is a vector of the “*Candidatus* Liberibacter” pathogen that is responsible for causing the lethal disease huanglongbing (HLB). *Diaphorina citri* colonizes citrus trees during the flushing periods of spring, summer and autumn (Qureshi *et al*., 2014). Citrus growers tend to apply insecticides (mostly systemic; Insecticide Resistance Action Committee, 2020) around 12 times per year against *D. citri* (Monzo & Stansly, 2017). Some of these insecticides, such as neonicotinoids, can remain in citrus trees for more than 1 year (Byrne *et al*., 2014); hence, while feeding on treated plants, numerous hemipterans can excrete honeydew that contains one or several systemic insecticides.

### (3) Horticultural crops

Brassicas such as cauliflower (*Brassica oleracea* var. *botrytis* L.), broccoli (*Brassica oleracea* var*. italica* L.), cabbage (*Brassica oleracea* var*. capitata* L.) or kale (*Brassica oleracea* var*. sabellica* L.) represent 3.8 Mha worldwide. In the EU and USA, these crops are grown on 278234 and 86194 ha, respectively (FAOSTAT, 2018). These crops are harvested before they flower; therefore, depending on the surrounding landscape, beneficial insects active in these crops may utilize honeydew excreted by aphids or whiteflies during the entire cropping period. In one study, 80% of *Cotesia glomerata* (L.) and 55% of *Microplitis mediator* (Haliday) parasitic wasps collected in cabbage fields had fed on honeydew, and only 16% of the *C. glomerata* collected in cabbage fields with flowering borders had fed exclusively on nectar (Wäckers & Steppuhn, 2003). Planting seeds coated with neonicotinoids in brassica crops has been discussed in the EU and the UK because brassicas are harvested before the flowering period and therefore, these crops do not pose risk to pollinators *via* contamination of nectar (European Commission, 2021; Government UK, 2021). However, where they are tolerant to insecticides, aphids and whiteflies can colonize seed‐coated *Brassica* plants at any plant growth stage and are likely to produce insecticide‐contaminated honeydew. The same situation might occur with fipronil. This phenylpyrazole was initially not considered a systemic insecticide, but some uptake by plants occurs (European Food Safety Authority, 2013*c*), especially if commercial formulations contain additional substances that alter its systemic properties (Dieckmann *et al*., 2010*a*,*b*,*c*; Bonmatin *et al*., 2015). It has been demonstrated recently that fipronil has sublethal effects on herbivorous insects that feed on brassicas grown from coated seeds (Gols, WallisDeVries & van Loon, 2020). Therefore, it might be also excreted by hemipterans. Fipronil is currently not allowed to be used in the EU. In the USA, however, fipronil is allowed for use on potatoes, although its applications are limited to Special Local Needs (FIFRA section 24c) (United States Department of Agriculture, 2014) because of potential environmental hazards (Tingle *et al*., 2003; Al‐Badran *et al*., 2018; Knodel & Shrestha, 2018). Horticultural crops such as tomatoes (*Solanum lycopersicum* L.), cucumbers (*Cucumis sativus* L.), aubergines (*Solanum melogena* L.), courgettes (*Cucurbita pepo* L.), etc. are important crops in the USA and the EU. For example, tomatoes are planted on 4924941 ha worldwide. In the EU and the USA, this crop is planted on 239724 and 130270 ha, respectively (FAOSTAT, 2018). Tomato flowers do not contain nectar and thus honeydew might be an important carbohydrate source for beneficial insects foraging in tomato fields (Fig. 5). In the EU, one of the most common systemic insecticides is chlorantraniliprole, which is used against the key pest *Tuta absoluta* (Meyrick) (Lepidoptera: Gelechiidae) (Biondi *et al*., 2018). Other systemic insecticides such as spirotetramat, sulfoxaflor, flonicamid or acetamiprid are used against whiteflies [*B. tabaci* and *Trialeurodes vaporariorum* (Westwood)] and/or aphids [*A. gossypii* or *Macrosiphum euphorbiae* (Thomas)] (Castañé, van der Blom & Nicot, 2020; Insecticide Resistance Action Committee, 2020). In addition, the neonicotinoids imidacloprid, clothianidin and thiamethoxam can be used in greenhouses against the above‐mentioned pests (European Commission, 2018a, 2018b, 2018c). In the USA, similar insecticides are allowed, in addition to pymetrozine, OPs or CMs (Donley, 2019). As a result, we expect hemipterans in tomato crops to excrete honeydew contaminated with neonicotinoids, sulfoximines, spirotetramat, flonicamid, pymetrozine, CMs, or OPs for at least short periods of time (a, d in Fig. 3). In addition, whiteflies and aphids, which are tolerant to chlorantraniliprole (Barrania & Abou‐Taleb, 2014), might excrete honeydew containing chlorantraniliprole until residues degrade in the plant (c in Fig. 3).

## VI HONEYDEW‐PRODUCERS AS INVASIVE PESTS

As a result of globalization, arthropod pests are increasingly invading new regions worldwide (Seebens *et al*., 2017). Many of these species excrete honeydew. As an example, the European and Mediterranean Plant Protection Organization (EPPO) has listed 39 invasive honeydew‐producing species that may arrive soon or have recently arrived in Europe (Table S3). These honeydew‐producing pests will continue to be treated with systemic insecticides until biological control can be established (Monzo & Stansly, 2017; Frank & Tooker, 2020; GIP Cítricos, 2021). For example, the mealybug *Delottococcus aberiae* De Lotto, which recently invaded the Mediterranean citrus area from South Africa (Beltra *et al*., 2015), is controlled with the systemic insecticides sulfoxaflor, acetamiprid or spirotetramat (GIP Cítricos, 2021). Similarly, in the USA, neonicotinoids are commonly applied against the Asian citrus psyllid *D. citri* in citrus, the soybean aphid *A. glycines* in soybean, or the polyphagous pest *Lycorma delicatula* (White) (Hemiptera: Fulgoridae) (Monzo & Stansly, 2017; Leach *et al*., 2019; Frank & Tooker, 2020). Applications with systemic insecticides, and excretion of honeydew contaminated with these insecticides, are likely to increase with continuing introductions of exotic and invasive pest species in future years (Frank & Tooker, 2020). New strategies to control invasive pests while reducing the application of systemic insecticides are clearly needed.

## VII CONCLUSIONS

1. Beneficial insects such as pollinators, parasitic wasps and predators can be exposed to honeydew contaminated with insecticides. Other plant‐derived food sources such as nectar, extrafloral nectar or guttation are important routes of insecticide exposure, but their availability tends to be restricted to brief flowering periods (for nectar), to a few crop plant species (those with extrafloral nectaries), or to specific climatic conditions (for guttation). By contrast, honeydew can be available during most of the growing season and for many crop species.
2. We suggest that systemic insecticides are likely to contaminate honeydew and describe several pathways through which this may take place.
3. Among hemipteran families, mealybugs, aphids and psyllids may excrete honeydew contaminated with systemic insecticides more often than whiteflies because the former families feed on both phloem and xylem.
4. Among insecticide groups, we suggest that neonicotinoids are the most likely to reach honeydew and negatively affect beneficial insects due to their high persistence in soil, water and plants, their high water solubility and high toxicity. Other insecticides that have lower persistence or toxicity, such as flonicamid or spirotetramat, are less likely to affect beneficial insects *via* honeydew.
5. We highlight valuable crop species for the EU and the USA that are commonly infested with hemipterans, and are treated with different systemic insecticides with likely impacts on beneficial insects that feed on honeydew. These concerns can be extrapolated to crop species in other parts of the world that are infested with hemipteran species.
6. This perspective paper broadcasts this route of exposure to environmental protection agencies and integrated pest management programs that regulate the use of systemic insecticides. We also recommend restricting the use of highly water‐soluble systemic insecticides that are persistent in the environment and those that have a broad‐spectrum activity to avoid non‐target impacts on beneficial insects through honeydew, and other routes of exposure.
7. This review describes how honeydew may play a role in insect declines when it is contaminated with systemic insecticides. Agricultural landscapes are increasingly monocultures, which are commonly infested by hemipterans and treated with systemic insecticides that likely reach honeydew. Honeydew is a hidden driver of direct and indirect interactions among insects that is likely to be affecting the population dynamics of herbivores, biological control agents, and pollinators (Evans & England, 1996; Ohgushi, 2008; Tena *et al*., 2016). Consequently, if honeydew is contaminated with insecticides, key interactions can be disrupted, altering trophic chains and ultimately, contributing to population declines of insects that feed on contaminated honeydew.

## Supporting information


**Table S1**. Current status of some systemic insecticides in Europe and the USA, and examples of resistant honeydew producers.
**Table S2**. Physiochemical properties and toxicity of systemic insecticides to honey bees.
**Table S3**. Honeydew‐producing invasive species from the European and Mediterranean Plant Protection Organisation (EPPO) lists.
