# Supplementary material for: Insecticide‐contaminated honeydew: risks for beneficial insects
Source: Biol Rev Camb Philos Soc. 2021 Nov 21;97(2):664–78. doi: 10.1111/brv.12817 (PMC9299500; doi:10.1111/brv.12817)
Supplement: Supplementary file 1 — Table S1. Current status of some systemic insecticides in Europe and the USA, and examples of resistant honeydew producers. Table S2. Physiochemical properties and toxicity of systemic insecticides to honey bees. Table S3. Honeydew‐producing invasive species from the European and Mediterranean Plant Protection Organisation (EPPO) lists. [file BRV-97-664-s001.docx]

# SUPPORTING INFORMATION

**Insecticide-contaminated honeydew: risks for beneficial insects in agriculture**

Miguel Calvo-Agudo^12^, John F. Tooker, Marcel Dicke and Alejandro Tena

*Corresponding author: Miguel Calvo Agudo. Email address: miguel.calvo.agudo1991@gmail.com

**This PDF file includes:**

Tables S1, S2, and S3

References

**Table S1.** Current status of some systemic insecticides in Europe and the USA and examples of resistant honeydew producers.

| **Insecticide** | **Current status in Europe** | **Current status in USA** | **Target pests** | **Examples of tolerant (blue), field resistant (red), and laboratory resistant (green) honeydew producers** |
| --- | --- | --- | --- | --- |
| Aldicarb | No | No | Broad spectrum | *Aphis gossypii* Glover (O’Brien *et al.*, 1992), *Myzus persicae* (Sulzer) (Hurej & Peters, 1989), *Bemisia tabaci* (Gennadius) (El Kady & Devine, 2003) |
| Carbaryl | No | Yes | Broad spectrum | *Cacopsylla pyri L.* (Zwick & Fields, 1978), *Myzocallis coryli* (Goeze) (Aliniazee, 1983), *Nilaparva lugens* (Stal) (Kilin *et al*., 1981), *Laodelphax. striatellus* (Fallén) (International Rice Research Institute, 1984), *Nephotettix cincticeps* (Uhler) (International Rice Research Institute, 1984) *A. gossypii* (Ahmad & Iqbal Arif, 2008) |
| Carbofuran | No | No | Broad spectrum | *A. gossypii* (Shang *et al.*, 2011), *M. persicae* (McClanahan & Founk, 1983), *N. lugens* (Yoo *et al.*, 2002), *Aphis glycines* Matsumura (Xi *et al.*, 2015) |
| Ethiofencarb | No | No | Broad spectrum | *M. persicae* (Sawicki & Rice, 1978) |
| Methomyl | No | Yes^*^ | Broad spectrum | *Planococcus ficus* Signoret (Prabhaker *et al.*, 2012), *Sitobion avenae* Fabricius (Zhang *et al.*, 2017), *M. persicae* (Tang *et al.*, 2017), *A. gossypii* (Ahmad & Iqbal Arif, 2008), *A. gossypii* (Shang *et al.*, 2011), *B. tabaci* (Fernández *et al.*, 2009), *Trialeurodes vaporariorum* Westwood (Elhag & Horn, 1983), *N. cincticeps* (International Rice Research Institute, 1984) |
| Pirimicarb | Yes | Yes | Aphids | *S. avenae* (Chen *et al.*, 2007), *A. gossypii* (Moores *et al.*, 1996), *Aphis nasturtii* Kaltenbach (Marcić *et al.*, 2007), *A. pomi* (Tamaš *et al.*, 2015), *M. persicae* (Herron *et al.*, 1993), *Nasonovia ribisnigri* (Mosely) (Barber *et al.*, 1999) |
| Acephate | No | Yes | Broad spectrum | *A. glycines* (Xi *et al.*, 2015), *A. gossypii* (Shang *et al.*, 2011), *M. persicae* (Srigiriraju *et al.*, 2009), *B. tabaci* (Roy *et al.*, 2019), *T. vaporariorum* (Omer *et al.*, 1992), *Diaphorina citri* Kuwayama (Rao *et al.*, 2018) |
| Azamethiphos | No | No | Broad spectrum | *B. tabaci* (Erdogan *et al.*, 2008) |
| Dimethoate | No | Yes | Broad spectrum | *P. ficus* (Prabhaker *et al.*, 2012), *A. gossypii* (Ahmad & Iqbal Arif, 2008), *Aphis pomi* de Geer (Tamaš *et al.*, 2015), *M. persicae* (Herron & Rophail, 1994), *Brevicoryne brassicae* (L.) (Radja *et al.*, 2019), *B. tabaci* (Ahmad *et al.*, 2002), *N. cincticeps* (International Rice Research Institute, 1984) |
| Methamidophos | No | No | Broad spectrum | *A. gossypii* (Moores *et al.*, 1996), *B. tabaci* (Pérez *et al.*, 2000), *M. persicae* (Herron *et al.*, 1993) |
| Malathion | Yes | Yes | Broad spectrum | *A. gossypii* (Shang *et al.*, 2011), *T. vaporariorum* (Elhag & Horn, 1983), *P. citri* (Serghiou, 1983), *P. pyricola* (Pielou & Downing, 1960), *L. striatellus* (International Rice Research Institute, 1984), ﻿*N. cincticeps* (International Rice Research Institute, 1984), *N. lugens* (International Rice Research Institute, 1984) |
| Vamidothion | No | No | Broad spectrum | *Eriosoma lanigerum* (Hausmann) (Pringle *et al.*, 1994), *N. cincticeps* (International Rice Research Institute, 1984) |
| Dicrotophos | No | Yes | Broad spectrum | *B. tabaci* (Dittrich *et al.*, 1986), *A. gossypii* (Grafton-Cardwell *et al.*, 1992), *M. persicae* (O’Brien *et al.*, 1992), *T. vaporariorum* (Omer *et al.*, 1992) |
| Dichlorvos/ DDVP | No | Yes | Broad spectrum | *T. vaporariorum* (Elhag & Horn, 1983), *B. tabaci* (He *et al.*, 2007), *A. gossypii* (Owusu *et al.*, 1996), *M. persicae* (Sudderuddin, 1973) |
| Ethiprole | No | No | Broad spectrum | *N. lugens* (Punyawattoe *et al.*, 2013) |
| Fipronil | No | Yes^**^ | Broad spectrum | *N. lugens* (Ling *et al.*, 2009), *B. tabaci* (Kang *et al.*, 2006) |
| Acetamiprid | Yes | Yes | Broad spectrum | *A. gossypii* (Herron & Wilson, 2011), *Phenacoccus solenopsis* Tinsley (Afzal *et al.*, 2015), *M. persicae* (Tang *et al.*, 2017), *B. tabaci* (He *et al.*, 2007) |
| Clothianidin | Yes^***^ | Yes | Broad spectrum | *M. persicae* (Foster *et al.*, 2008), *N. lugens* (Liao *et al.*, 2018), *A. gossypii* (Herron & Wilson, 2011) |
| Dinotefuran | No | Yes | Broad spectrum | *M. persicae* (Foster *et al.*, 2008), *N. lugens* (Liao *et al.*, 2018), *B. tabaci* (Roy *et al.*, 2019) |
| Imidacloprid | Yes^***^ | Yes | Broad spectrum | *P. ficus* (Prabhaker *et al.*, 2012), *S. avenae* (Zhang *et al.*, 2017), *A. gossypii* (Ma *et al.*, 2019), *M. persicae* (Tang *et al.*, 2017), *Myzus nicotianae* (Blackman) (Devine *et al.*, 1996), *B. tabaci* (Cahill *et al.*, 1996), *N. lugens* (Liao *et al.*, 2018), *B. argentifolii* (Prabhaker *et al.*, 1997), *T. vaporariorum* (Pappas *et al.*, 2013), *D. citri* (Rao *et al.*, 2018), *P. solenopsis* (Rezk *et al.*, 2019) |
| Nitenpyram | No |  | Broad spectrum | *A. gossypii* (Shi *et al.*, 2011), *B. tabaci* (Yao *et al.*, 2017), *N. lugens* (Liao *et al.*, 2018), *M. persicae* (Foster *et al.*, 2003), *L. striatellus* (Ban *et al.*, 2013) |
| Thiamethoxam | Yes^***^ | Yes | Broad spectrum | *B. tabaci* (Elbert & Nauen, 2000), *M. persicae* (Foster *et al.*, 2008), *D. citri* (Rao *et al.*, 2018), *A. gossypii* (Herron & Wilson, 2011), *N. lugens* (Liao *et al.*, 2018), *P. solenopsis* (Rezk *et al.*, 2019), *L. striatellus* (Ban *et al.*, 2013) |
| Thiacloprid | No | Yes | Broad spectrum | *A. gossypii* (Shi *et al.*, 2011), *B. tabaci* (Basit *et al.*, 2011), *T. vaporariorum* (Pappas *et al.*, 2013), *P. solenopsis* (Ahmad & Akhtar, 2016) |
| Sulfoxaflor | Yes | Yes | Hemipterans | *N. lugens* (Liao *et al.*, 2018), *B. tabaci* (Yao *et al.*, 2017), *A. gossypii* (Ma *et al.*, 2019), *M. persicae* (Z.H. Wang *et al.*, 2018)*.* |
| Flupyradifurone | Yes | Yes | Hemipterans | A. *gossypii* (Ma *et al.*, 2019) |
| Pymetrozine | No | Yes | Beetles, aphids, whiteflies | *L. striatellus* (Ban *et al.*, 2013), *B. tabaci* (Yao *et al.*, 2017), *P. citri* (Calvo-Agudo *et al.*, 2020), *F. dasylirii* (Barbosa *et al.*, 2018), *P. solenopsis* (Rezk *et al.*, 2019), *Rhopalosiphum maidis* (Fitch) (Tang *et al.*, 2013), *Aphis craccivora* Koch (Tang *et al.*, 2013) |
| Cartap hydrochloride | No | No | Aphids, whiteflies, jassids, thrips, mites | *B. tabaci* (Wang *et al.*, 2009), *R. maidis* (Tang *et al.*, 2013), *A. craccivora* (Tang *et al.*, 2013) |
| Thiocyclam | No | No | Thrips, hemipterans, lepidopterans, mites | *P. solenopsis* (Ahmad & Akhtar, 2016) |
| Spirotetramat | Yes | Yes | Hemipterans, thrips, mites | *A. gossypii* (Pan *et al.*, 2015), *P. solenopsis* (Ejaz & Ali Shad, 2017), *B. tabaci* (Bielza *et al.*, 2019) |
| Chlorantraniliprole | Yes | Yes | Lepidopterans, coleopterans and some dipterans | *P. solenopsis* (Nagrare *et al.*, 2016), *B. tabaci* (Barrania & Abou-Taleb, 2014), *A. gossypii* (Barrania & Abou-Taleb, 2014) |
| Cyantraniliprole | Yes | Yes | Lepidopterans, dipterans, whiteflies | *B. tabaci* (Yao *et al.*, 2017) |
| Flonicamid | Yes | Yes | Aphids, Thrips, Whiteflies | *A. gossypii* (IRAC, 2020), *B. tabaci* (Roy *et al.*, 2019), *P. citri* (Calvo-Agudo *et al*., 2020), *P. solenopsis* (Nagrare *et al.*, 2016) |

*Only allowed for soil application and only once every 3 years.

**Only allowed under a Special Local Need (FIFRA – section 24(c); United States Department of Agriculture, 2014).

***Only allowed for use in greenhouses (European Commission, 2018).

**Table S2.** Physiochemical properties of systemic insecticides.

| **Main group and primary site of action** | **Active ingredient subgroup** | **Group** | **Insecticide** | **Water solubility at 20ºC (mg/l)** | **Soil degradation (days) DT_50_ (laboratory 20 °C)** | **log K_ow_** | **pKa** | **Transport** | **Oral acute LD_50_ on honeybees** [**(μg/bee)**](https://es.wiktionary.org/wiki/%CE%BC) |
| --- | --- | --- | --- | --- | --- | --- | --- | --- | --- |
| Acetylcholinesterase (AChE) inhibitors | Carbamates | 1A | Aldicarb | 4,930 | 2.4 | 1.15 | ND | xylem | > 0.16 |
|  |  |  | Carbaryl | 9.1 | 16 | 2.36 | 10.4 | xylem | > 0.21 |
|  |  |  | Carbofuran | 322 | 12.8 | 1.8 | ND | xylem | 0.05 |
|  |  |  | Ethiofencarb | 1,900 | – | 2.04 | – | xylem | 1.54* |
|  |  |  | Oxamyl | 148,100 | 5.3 | –0.44 | 2.11 | xylem and phloem | 0.38 |
|  |  |  | Methomyl | 55,000 | 6.97 | 0.09 | ND | xylem | 0.28 |
|  |  |  | Pirimicarb | 3,100 | 73.6 | 1.7 | 4.4 | xylem and phloem (optimum) | 4.0 |
| Acetylcholinesterase (AChE) inhibitors | Organophosphates | 1B | Acephate | 790,000 | – | –0.85 | 8.35 | xylem | > 0.23 |
|  |  |  | Azamethiphos | 1,100 | – | 1.05 |  | xylem | > 0.10* |
|  |  |  | Dimethoate | 25,900 | 2.5 | 0.75 | ND | xylem | 0.1 |
|  |  |  | Methamidophos | 200,000 | 4 | –0.79 |  | xylem and phloem | > 0.22 |
|  |  |  | Malathion | 148 | 0.17 | 2.75 | ND | xylem | 0.40 |
|  |  |  | Vamidothion | 4,000 | – | –4.21 |  | xylem and phloem | 0.56* |
|  |  |  | Dicrotophos | 1,000,000 | – | –0.5 |  | xylem and phloem | 0.068 |
|  |  |  | Dichlorvos/ DDVP | 18,000 | 2 | 1.9 | ND | xylem | 0.29 |
| γ-aminobutyric acid (GABA)-gated chloride channel blockers | Phenylpyrazoles | 2B | Ethiprole | – | – | 1.99 |  | xylem | – |
|  |  |  | Fipronil | 3.78 | 142 | 3.75 | ND | xylem | 0.00417 |
| Nicotinic acetylcholine receptor (nAChR) competitive modulators | Neonicotinoid | 4A | Acetamiprid | 2,950 | 1.6 | 0.8 | 0.7 | xylem and phloem (optimum) | 14.53 |
|  |  |  | Clothianidin | 340 | 545 | 0.90 | 11.1 | xylem | 0.004 |
|  |  |  | Dinotefuran | 39,830 | – | –0.55 | 12.6 | xylem and phloem | > 0.023* |
|  |  |  | Imidacloprid | 610 | 187 | 0.57 |  | xylem | 0.0037 |
|  |  |  | Nitenpyram | 590,000 | – | –0.66 | 3.1 | xylem and phloem (optimum) | 0.138* |
|  |  |  | Thiamethoxam | 4,100 | 121 | –0.13 | ND | xylem and phloem | 0.005 |
|  |  |  | Thiacloprid | 184 | 0.88 | 1.26 | ND | xylem | 17.32 |
| Nicotinic acetylcholine receptor (nAChR) competitive modulators | Sulfoximines | 4C | Sulfoxaflor | 568 | 2.2 | 0.80 | ND | xylem | 0.146 |
| Nicotinic acetylcholine receptor (nAChR) competitive modulators | Butenolides | 4D | Flupyradifurone | 3,200 | 57.1 | 1.2 |  | xylem | 1.2 |
| Miscellaneous nonspecific (multi-site) inhibitors | Methyl isothiocyanate generators | 8F | Dazomet | 3,500 | 0.52 | 0.6 | ND | xylem | > 10 |
| Chordotonal organ TRPV channel modulators | Pyridine azomethine derivatives | 9B | Pymetrozine | 270 | 4.6 | –0.19 | 4.06 | xylem and phloem (optimum) | > 117 |
| Nicotinic acetylcholine receptor (nAChR) channel blockers | Nereistoxin analogues | 14 | Cartap hydrochloride | 200,000 | – | –0.95 |  | xylem and phloem | 10** |
|  |  |  | Thiocyclam | 84,000 | – | –0.07 | 3.95 | xylem and phloem (optimum) | – |
| Moulting disruptors in Diptera | Cyromazine | 17 | Cyromazine | 13,000 | 31.8 | 0.069 | 5.22 | xylem and phloem (optimum) | 186 |
| Ecdysone receptor agonists | Diacylhydrazines | 18 | Chromafenozide | 0.8 | 132 | 2.7 | 13.2 | xylem | > 133.2 |
| Inhibitors of acetyl CoA carboxylase | Tetronic and tetramic acid derivatives | 23 | Spirotetramat | 29.9 | 0.19 | 2.51 | 10.7 | xylem | > 107.3 |
| Ryanodine receptor modulators | Diamides | 28 | Chlorantraniliprole | 0.88 | 597 | 2.86 | 10.8 | xylem | > 104.1 |
|  |  | 28 | Cyantraniliprole | 14.2 | 34.4 | 2.02 | 8.8 | xylem | > 0.09* |
|  |  | 28 | Cyclaniliprole | 0.15 | 1161 | 2.7 | 8.6 | xylem | 0.66 |
| Chordotonal organ modulators: undefined target site | Flonicamid | 29 | Flonicamid | 5,200 | 1.1 | –0.24 | 11.6 | xylem and phloem | > 100 |
| Compounds of unknown or uncertain mode of action | Benzoximate | UN | Benzoximate | – | – | 2.4 | ND | xylem | – |

DT_50_, rate of degradation of pesticide or half-life; K_ow_, octanol/water-partition coefficient; LD_50_, median lethal dose; ND: no dissociation; pKa, dissociation constant.

Data obtained from Pesticide Properties Database of the University of Hertfordshire (link: http://sitem.herts.ac.uk/aeru/ppdb/en/atoz_insect.htm).

*LD_50_ for acute contact toxicity.

**LD_50_ for an unknown mode of exposure.

**Table S3.** Honeydew-producing invasive species from the European and Mediterranean Plant Protection Organization (EPPO) lists.

| **Honeydew-producer species** | **Family** | | **EPPO list*** | **Host range** | **System affected**** |
| --- | --- | --- | --- | --- | --- |
| *Aleurocanthus woglumi* | Aleyrodidae | A1 | | Polyphagous | 1 |
| *Bactericera cockerelli* (vector of *Candidatus* Liberibacter solanacearum) | Triozidae | A1 | | Oligophagous | 1 |
| *Diaphorina citri* (vector of *Candidatus* Liberibacter asiaticus) | Liviidae | A1 | | Oligophagous | 1 |
| *Haplaxius crudus* (vector of *Candidatus* Phytoplasma palmae) | Cixiidae | A1 | | Oligophagous | 1 |
| *Homalodisca vitripennis* (vector of *Xylella fastidiosa*) | Cicadellidae | A1 | | Polyphagous | 1, 2, 3 |
| [*Lycorma delicatula*](https://gd.eppo.int/taxon/LYCMDE) | Fulgoridae | A1 | | Polyphagous | 1, 2, 3 |
| [*Margarodes prieskaensis*](https://gd.eppo.int/taxon/MARGPR) | Margaroridae | A1 | | Monophagous | 1 |
| [*Margarodes vitis*](https://gd.eppo.int/taxon/MARGVI) | Margaroridae | A1 | | Polyphagous | 1, 2, 3 |
| [*Margarodes vredendalensis*](https://gd.eppo.int/taxon/MARGVR) | Margaroridae | A1 | | Monophagous | 1, 2, 3 |
| [*Ripersiella hibisci*](https://gd.eppo.int/taxon/RHIOHI) | Pseudococcidae | A1 | | Polyphagous | 1, 2, 3 |
| [*Aleurocanthus spiniferus*](https://gd.eppo.int/taxon/ALECSN) | Aleyrodidae | A2 | | Polyphagous | 1, 2, 3 |
| *Bemisia tabaci* | Aleyrodidae | A2 | | Polyphagous | 1, 2, 3 |
| [*Daktulosphaira vitifoliae*](https://gd.eppo.int/taxon/VITEVI) | Phylloxeridae | A2 | | Monophagous | 1 |
| [*Maconellicoccus hirsutus*](https://gd.eppo.int/taxon/PHENHI) | Pseudococcidae | A2 | | Polyphagous | 1, 2, 3 |
| [*Trioza erytreae*](https://gd.eppo.int/taxon/TRIZER) (vector of *Candidatus* Liberibacter africanus) | Triozidae | A2 | | Oligophagous | 1, 2, 3 |
| [*Toxoptera citricidus*](https://gd.eppo.int/taxon/TOXOCI) | Aphididae | A2 | | Polyphagous | 1, 2, 3 |
| *Crisicoccus pini* | Pseudococcidae | Alert list | | Monophagous | 2 |
| *Toumeyella parvicornis* | Coccidae | Alert list | | Monophagous | 2 |
| [*Acizzia jamatonica*](https://gd.eppo.int/taxon/ACIZJA) | Psyllidae | Previously listed pests | | Monophagous | 3 |
| *Aleurodicus dispersus* | Aleyrodidae | Previously listed pests | | Polyphagous | 1, 2, 3 |
| *Aleurothrixus trachoides* | Aleyrodidae | Previously listed pests | | Polyphagous | 1, 2, 3 |
| *Cacopsylla fulguralis* | Psyllidae | Previously listed pests | | Monophagous | 3 |
| *Ceroplastes ceriferus* | Coccidae | Previously listed pests | | Polyphagous | 1, 2, 3 |
| *Corythucha arcuata* | Tingidae | Previously listed pests | | Polyphagous | 3 |
| *Homalodisca vitripennis* | Cicadellidae | Previously listed pests | | Polyphagous | 1, 2, 3 |
| *Aleurodicus floccissimus* | Aleyrodidae | Previously listed pests | | Polyphagous | 1, 2, 3 |
| *Lycorma delicatula* | Fulgoridae | Previously listed pests | | Polyphagous | 1, 2, 3 |
| *Ctenarytaina spatulata* | Aphalaridae | Previously listed pests | | Monophagous | 2 |
| *Glycaspis brimblecombei* | Aphalaridae | Previously listed pests | | Monophagous | 2 |
| *Marchalina hellenica* | Margaroridae | Previously listed pests | | Oligophagous | 2 |
| *Phenacoccus gossypi* | Pseudococcidae | Previously listed pests | | Polyphagous | 1, 2, 3 |
| *Rhizoecus americanus* | Pseudococcidae | Previously listed pests | | Polyphagous | 1, 2, 3 |
| *Singhiella simplex* | Aleyrodidae | Previously listed pests | | Oligophagous | 3 |
| *Stephanitis pyrioides* | Tingidae | Previously listed pests | | Oligophagous | 3 |
| *Stephanitis takeyai* | Tingidae | Previously listed pests | | Oligophagous | 3 |
| *Tetraleurodes perseae* | Aleyrodidae | Previously listed pests | | Monophagous | 1 |
| *Trialeurodes ricini* | Aleyrodidae | Previously listed pests | | Oligophagous | 1,3 |

*The EPPO aims to protect plant health in agriculture by creating lists of invasive species that should be quarantined. List A1 contains quarantine pests that have not arrived in the member countries of EPPO yet, whereas List A2 contain pests that are locally present within the EPPO region. In addition, EPPO creates Alert Lists to draw attention of EPPO member countries to pests that may represent a risk (early warnings). For species on the Alert Lists, pest risk analyses are carried out and depending on their results these pests are transferred to A1 and A2 lists, or removed after 3 years (‘previously listed pests’). This table includes all hemipteran species that excrete honeydew from all EPPO lists.

**System affected: 1 = agriculture; 2 = forestry; 3 = other uses (i.e. ornamental, pastures, etc.).

**References**

Afzal, M.B.S., Shad, S.A., Abbas, N., Ayyaz, M. & Walker, W.B. (2015). Cross-resistance, the stability of acetamiprid resistance and its effect on the biological parameters of cotton mealybug, *Phenacoccus solenopsis* (Homoptera: Pseudococcidae), in Pakistan. *Pest Management Science* **71**, 151–158.

Ahmad, M. & Akhtar, S. (2016). Development of resistance to insecticides in the invasive mealybug *Phenacoccus solenopsis* (Hemiptera: Pseudococcidae) in Pakistan. *Crop Protection* **88**, 96–102.

Ahmad, M., Arif, M.I., Ahmad, Z. & Denholm, I. (2002). Cotton whitefly (*Bemisia tabaci*) resistance to organophosphate andpyrethroid insecticides in pakistan. *Pest Management Science* **58**, 203–208.

Ahmad, M. & Iqbal Arif, M. (2008). Susceptibility of Pakistani populations of cotton aphid *Aphis gossypii* (Homoptera: Aphididae) to endosulfan, organophosphorus and carbamate insecticides. *Crop Protection* **27**, 523–531.

Aliniazee, M.T. (1983). Carbaryl Resistance in the filbert aphid (Homoptera: Aphididae). *Journal of Economic Entomology* **76**, 1002–1004.

Ban, L., Zhang, S., Huang, Z., He, Y., Peng, Y. & Gao, C. (2013). Resistance monitoring and assessment of resistance risk to pymetrozine in *Laodelphax striatellus* (Hemiptera: Delphacidae). *Journal of Economic Entomology* **105**, 2129–2135.

Barber, M.D., Moores, G.D., Tatchell, G.M., Vice, W.E. & Denholm, I. (1999). Insecticide resistance in the currant–lettuce aphid, *Nasonovia ribisnigri* (Hemiptera: Aphididae) in the UK. *Bulletin of Entomological Research* **89**, 17–23.

Barbosa, P.R.R., Oliveira, M.D., Barros, E.M., Michaud, J.P. & Torres, J.B. (2018). Differential impacts of six insecticides on a mealybug and its coccinellid predator. *Ecotoxicology and Environmental Safety* **147**, 963–971.

Barrania, A.A. & Abou-Taleb, H.K. (2014). Field efficiency of some insecticide treatments against whitefly, *Bemisia tabaci*, cotton aphid, *Aphis gossypii* and their associated predator, *Chrysopa vulgaris*, in cotton plants. *Alex. J. Agric. Res* **59**, 105–111.

Basit, M., Sayyed, A.H., Saleem, M.A. & Saeed, S. (2011). Cross-resistance, inheritance and stability of resistance to acetamiprid in cotton whitefly, *Bemisia tabaci* Genn (Hemiptera: Aleyrodidae). *Crop Protection* **30**, 705–712.

Bielza, P., Moreno, I., Belando, A., Grávalos, C., Izquierdo, J. & Nauen, R. (2019). Spiromesifen and spirotetramat resistance in field populations of *Bemisia tabaci* Gennadius in Spain. *Pest Management Science* **75**, 45–52.

Cahill, M.R., Macey, M.G., Dawson, J.R. & Newland, A.C. (1996). Platelet surface activation antigen expression at baseline and during elective angioplasty in patients with mild to moderate coronary artery disease. *Blood Coagulation and Fibrinolysis* **7**, 165–168.

Calvo-Agudo, M., González-Cabrera, J., Sadutto, D., Picó, Y., Urbaneja, A., Dicke, M. & Tena, A. (2020). IPM-recommended insecticides harm beneficial insects through contaminated honeydew. *Environmental Pollution* **267**, 115581.

Chen, M., Han, Z., Qiao, X. & Qu, M. (2007). Resistance mechanisms and associated mutations in acetylcholinesterase genes in *Sitobion avenae* (Fabricius). *Pesticide Biochemistry and Physiology* **87**, 189–195.

Devine, G.J., Harling, Z.K., Scarr, A.W. & Devonshire, A.L. (1996). Lethal and sublethal effects of imidacloprid on nicotine-tolerant *Myzus nicotianae* and *Myzus persicae*. *Pesticide Science* **48**, 57–62.

Dittrich, V., Hassan, S.O. & Ernst, G.H. (1986). Development of a new primary pest of cotton in the Sudan: *Bemisia tabaci*, the whitefly. *Agriculture*, *ecosystems & environment* **17**, 137–142.

Ejaz, M. & Ali Shad, S. (2017). Spirotetramat resistance selected in the *Phenacoccus solenopsis* (Homoptera: Pseudococcidae): cross-resistance patterns, stability, and fitness costs analysis. *Journal of economic entomology* **110**, 1226–1234.

Elbert, A. & Nauen, R. (2000). Resistance of *Bemisia tabaci* (Homoptera: Aleyrodidae) to insecticides in southern Spain with special reference to neonicotinoids. *Pest Management Science* **56**, 60–64.

Elhag, E.A. & Horn, D.J. (1983). Resistance of greenhouse whitefly (Homoptera: Aleyrodidae) to insecticides in selected Ohio greenhouses. *Journal of Economic Entomology* **76**, 945–948.

El Kady, H. & Devine, G.J. (2003). Insecticide resistance in Egyptian populations of the cotton whitefly, *Bemisia tabaci* (Hemiptera: Aleyrodidae). *Pest Management Science* **59**, 865–871.

Erdogan, C., Moores, G.D., Oktay Gurkan, M., Gorman, K.J. & Denholm, I. (2008). Insecticide resistance and biotype status of populations of the tobacco whitefly *Bemisia tabaci* (Hemiptera: Aleyrodidae) from Turkey. *Crop Protection* **27**, 600–605.

European Commission (2018). Implementing Commission Regulations 2018/783/EC, 2018/784/EC, 2018/785/EC. L 132, 30.5.2018. *Official Journal of the European Union*.

Fernández, E., Grávalos, C., Haro, P.J., Cifuentes, D. & Bielza, P. (2009). Insecticide resistance status of *Bemisia tabaci* Q-biotype in south-eastern Spain. *Pest Management Science* **65**, 885–891.

Foster, S.P., Cox, D., Oliphant, L., Mitchinson, S. & Denholm, I. (2008). Correlated responses to neonicotinoid insecticides in clones of the peach‐potato aphid, *Myzus persicae* (Hemiptera: Aphididae). *Pest management science* **64**, 1111–1114.

Foster, S.P., Denholm, I. & Thompson, R. (2003). Variation in response to neonicotinoid insecticides in peach-potato aphids, *Myzus persicae* (Hemiptera: Aphididae). *Pest Management Science* **59**, 166–173.

Grafton-Cardwell, E.E., Leigh, T.F., Bentley, W.J. & Goodell, P.B. (1992) Cotton aphids have become resistant to commonly used pesticides. *California Agriculture* **46**, 4–7.

He, Y.-X., Weng, Q.-Y., Huang, J., Liang, Z.-S., Lin, G.-J. & Wu, D.-D. (2007). Insecticide resistance of *Bemisia tabaci* field populations. *Chinese Journal of Applied Ecology* **18**, 1578—1582.

Herron, G.A., Gibson, T.S. & Horwood, M.A. (1993). Insecticide resistance in *Myzus persicae* (Sulzer) (Hemiptera: Aphididae) in Southeastern Australia. *Australian Journal of Entomology* **32**, 23–27.

Herron, G.A. & Rophail, J. (1994). Insecticide Resistance Detected in *Myzus persicae* (Sulzer) (Hemiptera: Aphididae) from New South Wales Cotton. *Australian Journal of Entomology* **33**, 263–264.

Herron, G.A. & Wilson, L.J. (2011). Neonicotinoid resistance in *Aphis gossypii* Glover (Aphididae: Hemiptera) from Australian cotton. *Australian Journal of Entomology* **50**, 93–98.

Hurej, M. & Peters, D. (1989). Sublethal effects of aldicarb on the behaviour of *Aphis fabae* and two clones of *Myzus persicae* and on the transmission of beet mosaic virus by these aphids. *Entomologia Experimentalis et Applicata* **50**, 81–86.

Insecticide Resistance Action Committee (2020). Modes of Action (MoA) Classification | IRAC. Https://www.irac-online.org/modes-of-action/.

International Rice Research Institute (1984). Present and future directions of chemical control research. In *Proceeding of the FAO/IRRI workshop on Judicious and efficient use of insecticides on rice* pp. 80–81.

Kang, C.Y., Wu, G. & Miyata, T. (2006). Synergism of enzyme inhibitors and mechanisms of insecticide resistance in *Bemisia tabaci* (Gennadius) (Hom., Aleyrodidae). *Journal of Applied Entomology* **130**, 377–385.

Kilin, D., Nagata, T. & Masuda, T. (1981). Development of carbamate resistance in the brown planthopper *Nilaparvata lugens* Stal (Homoptera: Delphacidae). *Chemical and Pharmaceutical Bulletin* **16**, 1–6.

Liao, X., Jin, R., Zhang, X., Ali, E., Mao, K., Xu, P., Li, J. & Wan, H. (2018). Characterization of sulfoxaflor resistance in the brown planthopper, *Nilaparvata lugens* (Stål). *Pest Management Science* **75**, 1646–1654.

Ling, S., Zhang, J., HU, L. & Zhang, R. (2009). Effect of fipronil on the reproduction, feeding, and relative fitness of brown planthopper, *Nilaparvata lugens*. *Applied Entomology and Zoology* **44**, 543–548.

Ma, K., Tang, Q., Zhang, B., Liang, P., Wang, B. & Gao, X. (2019). Overexpression of multiple cytochrome P450 genes associated with sulfoxaflor resistance in *Aphis gossypii* Glover. *Pesticide Biochemistry and Physiology* **157**, 204–210. Elsevier.

Marcić, D., Kljajić, P., Krnjajić, S. & Perić, I. (2007). Studies of the efficacy of insecticides against pepper-infesting aphids (Aphididae). *Acta Horticulturae* **729**, 483–487.

McClanahan, R.J. & Founk, J. (1983). Toxicity of insecticides to the green peach aphid (Homoptera: Aphididae) in laboratory and field tests. *Journal of Economic Entomology* **76**, 899–905.

Moores, G.D., Gao, X., Denholm, I. & Devonshire, A.L. (1996). Characterisation of insensitive acetylcholinesterase in insecticide-resistant cotton aphids, *Aphis gossypii* Glover (Homoptera: Aphididae). *Pesticide Biochemistry and Physiology* **56**, 102–110.

Nagrare, V.S., Kranthi, S., Kranthi, K.R., Naik, V.C.B., Deshmukh, V., Naikwadi, B. & Dahekar, A. (2016). Relative toxicity of insecticides against cotton mealybug *Phenacoccus solenopsis* Tinsley (Hemiptera:Pseudococcidae) and its fortuous parasitod *Aenasius bambawalei* Hayat (Hymenoptera: Encyrtidae). *Journal of Applied and Natural Science* **8**, 987–994.

O’Brien, P.J., Abdel-Aal, Y.A., Ottea, J.A. & Graves, J.B. (1992). Relationship of insecticide resistance to carboxylesterases in *Aphis gossypii* (Homoptera: Aphididae) from Midsouth Cotton. *Journal of Economic Entomology* **85**, 651–657.

Omer, A.D., Leigh, T.F. & Granett, J. (1992). Insecticide resistance in field populations of greenhouse whitefly (Homoptera: Aleyrodidae) in the San Joaquin Valley (California) cotton cropping system. *Journal of Economic Entomology* **85**, 21–27.

Owusu, E.O., Horiike, M. & Hirano, C. (1996). Polyacrylamide gel electrophoretic assessments of esterases in cotton aphid (Homoptera: Aphididae) resistance to dichlorvos. *Journal of Economic Entomology* **89**, 302–306.

Pan, Y., Yang, C., Gao, X., Peng, T., Bi, R., Xi, J., Xin, X., Zhu, E., Wu, Y. & Shang, Q. (2015). Spirotetramat resistance adaption analysis of *Aphis gossypii* Glover by transcriptomic survey. *Pesticide Biochemistry and Physiology* **124**, 73–80. Elsevier Inc.

Pappas, M.L., Migkou, F. & Broufas, G.D. (2013). Incidence of resistance to neonicotinoid insecticides in greenhouse populations of the whitefly, *Trialeurodes vaporariorum* (Hemiptera: Aleyrodidae) from Greece. *Applied Entomology and Zoology* **48**, 373–378.

Pérez, C.J., Alvarado, P., Narváez, C., Miranda, F., Hernández, L., Vanegas, H., Hruska, A. & Shelton, A.M. (2000). Assessment of insecticide resistance in five insect pests attacking field and vegetable crops in Nicaragua. *Journal of Economic Entomology* **93**, 1779–1787.

Pielou, D.P. & Downing, R.S. (1960). Dimethoate, a systemic of low mammalian toxicity, as an orchard insecticide in British Columbia. *Journal of the Entomological Society of British Columbia* **57**, 52–57.

Prabhaker, N., Gispert, C. & Castle, S.J. (2012). Baseline susceptibility of *Planococcus ficus* (Hemiptera: Pseudococcidae) from California to select insecticides. *Journal of Economic Entomology* **105**, 1392–1400.

Prabhaker, N., Toscano, N.C., Castle, S.J. & Henneberry, T.J. (1997). Selection for imidacloprid resistance in silverleaf whiteflies from the imperial valley and development of a hydroponic bioassay for resistance monitoring. *Pesticide Science* **51**, 419–428.

Pringle, K.L., Giliomee, J.H. & Addison, M.F. (1994). Vamidothion tolerance in a strain of the woolly apple aphid, *Eriosoma lanigerum* (Hausmann) (Hemiptera: Aphididae). *African Entomology* **2**, 123–125.

Punyawattoe, P., Han, Z., Sriratanasak, W., Arunmit, S., Chaiwong, J. & Bullangpoti, V. (2013). Ethiprole resistance in *Nilaparvata lugens* (Hemiptera: Delphacidae): Possible mechanisms and cross-resistance. *Applied Entomology and Zoology* **48**, 205–211.

Radja, K.H., Mikani, A. & Mosallanejad, H. (2019). Biochemical resistance mechanisms to dimethoate in cabbage aphid *Brevicoryne brassicae* (L.) (Hom.: Aphididae). *Journal of Agricultural Science and Technology* **22**, 187–196.

Rao, C.N., George, A. & Rahangadale, S. (2018). Monitoring of resistance in field populations of *Scirtothrips dorsalis* (Thysanoptera: Thripidae) and *Diaphorina citri* (Hemiptera: Liviidae) to commonly used insecticides in citrus in Central India. *Journal of Economic Entomology* **112**, 324–328.

Rezk, M., Hassan, A.N.T., El-Deeb, M.F., Shaarawy, N. & Dewer, Y. (2019). The impact of insecticides on the cotton mealybug, Phenacoccus solenopsis (Tinsley): efficacy on potato, a new record of host plant in Egypt. *Journal of Plant Protection Research* **59**, 50–59.

Roy, D., Bhattacharjee, T., Biswas, A., Ghosh, A., Sarkar, S., Mondal, D. & Sarkar, P.K. (2019). Resistance monitoring for conventional and new chemistry insecticides on *Bemisia tabaci* genetic group Asia-I in major vegetable crops from India. *Phytoparasitica* **47**, 55–66.

Sawicki, R.M. & Rice, A.D. (1978). Response of susceptible and resistant peach‐potato aphids *Myzus persicae* (Sulz.) to insecticides in leaf‐dip bioassays. *Pesticide Science* **9**, 513–516.

Serghiou, C.S. (1983). The citrus mealybug, *Planococcus Citri* Risso ­ Carob Moth, *Ectomyelois Ceratoniae* Zeller, pest complex on grapefruit and its chemical control. In *Technical bulletin-Agricultural Research Institute*.

Shang, Q., Pan, Y., Fang, K., Xi, J. & Brennan, J.A. (2011). Biochemical characterization of acetylcholinesterase, cytochrome P450 and cross-resistance in an omethoate-resistant strain of *Aphis gossypii* Glover. *Crop Protection* **31**, 15–20.

Shi, X., Jiang, L., Wang, H., Qiao, K., Wang, D. & Wang, K. (2011). Toxicities and sublethal effects of seven neonicotinoid insecticides on survival, growth and reproduction of imidacloprid-resistant cotton aphid, *Aphis gossypii*. *Pest Management Science* **67**, 1528–1533.

Srigiriraju, L., Semtner, P.J., Anderson, T.D. & Bloomquist, J.R. (2009). Esterase-based resistance in the tobacco-adapted form of the green peach aphid, *Myzus persicae* (Sulzer) (Hemiptera: Aphididae) in the Eastern United States. *Archives of Insect Biochemistry and Physiology* **72**, 105–123.

Sudderuddin, K.I. (1973). Studies of insecticide resistance in *Myzus persicae* (Sulz.) (Hem., Aphididae). *Bulletin of Entomological Research* **62**, 533–539.

Tamaš, N., Dojnov, B., Margetić, A., Vujčić, M., Špirović, B., Miletić, N., Stević, M. & Vujčić, Z. (2015). Resistance to common organophosphate and carbamate insecticides in *Aphis pomi* (Hemiptera: Aphididae). *Fruits* **70**, 135–142.

Tang, L. De, Wu, J.H., Ali, S. & Ren, S.X. (2013). Establishment of baseline toxicity data to different insecticides for *Aphis craccivora* Koch and *Rhopalosiphum maidis* (Fitch) (Homoptera: Aphididae) by glass tube residual film technique. *Pakistan Journal of Zoology* **45**, 411–415.

Tang, Q.L., Ma, K.S., Hou, Y.M. & Gao, X.W. (2017). Monitoring insecticide resistance and diagnostics of resistance mechanisms in the green peach aphid, *Myzus persicae* (Sulzer) (Hemiptera: Aphididae) in China. *Pesticide Biochemistry and Physiology* **143**, 39–47.

United States Department of Agriculture (2014). Pesticide use in the imported fire ant program.

Wang, Z., Yao, M. & Wu, Y. (2009). Cross-resistance, inheritance and biochemical mechanisms of imidacloprid resistance in B-biotype *Bemisia tabaci*. *Pest Management Science* **65**, 1189–1194.

Wang, Z.H., Gong, Y.J., Chen, J.C., Su, X.C., Cao, L.J., Hoffmann, A.A. & Wei, S.J. (2018). Laboratory selection for resistance to sulfoxaflor and fitness costs in the green peach aphid *Myzus persicae*. *Journal of Asia-Pacific Entomology* **21**, 408–412.

Xi, J., Pan, Y., Bi, R., Gao, X., Chen, X., Peng, T., Zhang, M., Zhang, H., Hu, X. & Shang, Q. (2015). Elevated expression of esterase and cytochrome P450 are related with lambda-cyhalothrin resistance and lead to cross resistance in *Aphis glycines* Matsumura. *Pesticide Biochemistry and Physiology* **118**, 77–81. Elsevier Inc.

Yao, F.L., Zheng, Y., Huang, X.Y., Ding, X.L., Zhao, J.W., Desneux, N., He, Y.X. & Weng, Q.Y. (2017). Dynamics of Bemisia tabaci biotypes and insecticide resistance in Fujian province in China during 2005-2014. *Scientific Reports* **7**, 40803.

Yoo, J.K., Lee, S.W., Ahn, Y.J., Nagata, T. & Shono, T. (2002). Altered acetylcholinesterase as a resistance mechanism in the brown planthopper (Homoptera: Delphacidae), *Nilaparvata lugens* Stål. *Applied Entomology and Zoology* **37**, 37–41.

Zhang, L., Lu, H., Guo, K., Yao, S. & Cui, F. (2017). Insecticide resistance status and detoxification enzymes of wheat aphids *Sitobion avenae* and *Rhopalosiphum padi*. *Science China Life Sciences* **60**, 927–930.

Zwick, R.W. & Fields, G.J. (1978). Field and laboratory evaluations of fenvalerate against several insect and mite pests of apple and pear in Oregon. *Journal of Economic Entomology* **71**, 793–796.
